# Functional Characterization of the Nuclear Receptor Gene *SaE75* in the Grain Aphid, *Sitobion avenae*

**DOI:** 10.3390/insects14040383

**Published:** 2023-04-14

**Authors:** Haixia Zheng, Yi Yan, Guohua Wei, Austin Merchant, Yaxin Gu, Xuguo Zhou, Xun Zhu, Yunhui Zhang, Xiangrui Li

**Affiliations:** 1 College of Plant Protection, Shanxi Agricultural University, Taigu, Jinzhong 030800, China; 2 State Key Laboratory for Biology of Plant Diseases and Insect Pests, Institute of Plant Protection, Chinese Academy of Agricultural Sciences, Beijing 100193, China; 3 Department of Entomology, University of Kentucky, Lexington, KY 40546, USA; 4 College of Plant Science, Tibet Agriculture and Animal Husbandry University, Linzhi 860000, China

**Keywords:** *Sitobion avenae*, *SaE75*, ecdysone signaling pathway, 20E, molting

## Abstract

**Simple Summary:**

The English grain aphid, *Sitobion avenae*, is one of the major pests and disease vectors of wheat crops. In this study, *E75*, a key gene associated with ecdysone signal pathway, was identified and cloned from *S. avenae*. Functional characterization of *E75* confirmed its role in the regulation of growth, development, and molting process of aphids. This finding offers a potential molecular target for the subsequent development of genetic-based biopesticides for aphid control.

**Abstract:**

Ecdysteroid hormones are key regulators of insect development and metamorphosis. Ecdysone-inducible *E75*, a major component of insect ecdysone signaling pathway, has been well characterized in holometabolous insects, however, barely in hemimetabolous species. In this study, a total of four full-length *E75* cDNAs from the English grain aphid, *Sitobion avenae*, were identified, cloned, and characterized. The four *SaE75* cDNAs contained 3048, 2625, 2505, and 2179 bp open reading frames (ORF), encoding 1015, 874, 856, and 835 amino acids, respectively. Temporal expression profiles showed that *SaE75* expression was low in adult stages, while high in pseudo embryo and nymphal stages. *SaE75* was differentially expressed between winged and wingless morphs. RNAi-mediated suppression of *SaE75* led to substantial biological impacts, including mortality and molting defects. As for the pleiotropic effects on downstream ecdysone pathway genes, *SaHr3* (hormone receptor like in 46) was significantly up-regulated, while *Sabr-c* (broad-complex core protein gene) and *Saftz-f1* (*transcription factor 1*) were significantly down-regulated. These combined results not only shed light on the regulatory role of *E75* in the ecdysone signaling pathway, but also provide a potential novel target for the long-term sustainable management of *S. avenae*, a devastating global grain pest.

## 1. Introduction

There are over 5000 estimated aphid species [1], of which 450 feed on crops and 14 are considered serious agricultural pests [2]. Given their diversity, ecological and economical significance, aphids have emerged as model organisms to study phenotypic plasticity [3,4] and invasion biology [5]. The English grain aphid, *Sitobion avenae* (Fabricius), is a major pest of wheat that damages plants by sucking sap, which affects the grain filling stage; excreting honeydew, which affects plant photosynthesis; and transmitting barley yellow dwarf virus, which reduces wheat yield. Altogether, the damage caused by *S. avenae* results in great losses to agriculture and the economy every year in China [6,7]. Aphids show two different morphs, winged and wingless, which are associated with external environmental factors, such as temperature, light, and host nutrition [8]; the regulation of wing development genes [3]; and the influence of endocrine hormones [9,10].

The development and metamorphosis of insects is an important physiological process. Juvenile hormone (JH) and 20 Hydroxyecdysone (20E) jointly regulate the processes of insect development and metamorphosis [11]. 20E induces insect molting and metamorphosis, while JH inhibits insect development and metamorphosis. Precursor hormone ecdysone (E) is synthesized from dietary cholesterol or plant sterols in insect prothoracic glands. The E is then secreted into the body cavity and transformed into 20E in various peripheral tissues of the insect midgut and fat body. 20E combines with ecdysone receptor (EcR) and ultraspiracle protein (USP) to form a heterodimeric receptor that initiates the expression of downstream transcription factors involved in regulating 20E-mediated roles in growth, development, reproduction, molting, and metamorphosis [12]. The genes impacted via EcR/USP transcriptional activity are other nuclear receptors as well as “early” genes; then, for upregulation of a set of “early-late” genes, via ftz-f1, the signal will be passed on to the “late” genes required for successful molting/metamorphosis [13].

Nuclear receptors (NRSs) are ligand-dependent transcription factors that play an important role in individual growth and development, cell differentiation, and many other physiological and metabolic processes in vivo [14]. In insects, nuclear receptors generally have five domains: an A/B domain (trans-activation domain), C domain (DNA-binding domain, DBD), D domain (hinge domain) E domain (ligand-binding domain, LBD), and F domain (C-terminal domain) [15]. The nuclear receptor superfamily is divided into seven subfamilies, N0-N6, according to sequence homology. Both DBD and LBD domains exist in N1-N6, while only a DBD domain exists in N0 [16]. DBD and LBD are two highly conserved domains in the nuclear receptor family. DBD interacts with the reaction elements in the promoters of regulated genes, while LBD binds to the proteins of known ligands and is regulated by small molecular ligands. Based on the conservation of DBD and LBD, homologous NRSs have been detected in vertebrates, insects, and nematodes, and 18–19 NRSs have been identified in insects [17]. Therefore, NRSs can be identified through DBD and LBD. The different subtypes of nuclear receptor genes are mainly caused by differences in the A/B domain.

Ecdysone-induced protein 75 (E75) belongs to the nuclear receptor NR1 subfamily and is widely distributed across ecdysozoa taxa. *E75* is a key early response gene in the insect ecdysone signaling pathway, which affects ecdysteroid titer. *E75* are activated in response to EcR/USP heterodimer involved in activating the expression of “early-late” genes [18]. Four *E75* variants with different N-terminal regions have been identified in *Drosophila melanogaster* [19,20]. Mutation analysis of all *Drosophila DE75* subtypes showed that they were necessary for embryonic survival [18]. *E75* also plays an important role in individual growth and development in other animals and insects. After silencing *E75* of the prawn *Exopalaemon carinicauda*, molting rate was significantly reduced, and growth and development were delayed [21]. Silencing *LdE75* of the Colorado potato beetle, *Leptinotarsa decemlineata*, reduced 20E titer, affected the expression of genes involved in the ecdysone signaling pathway, and finally led to significant mortality due to difficulty in pupation [22]. RNAi-induced knockdown of *E75* in the silk moth *Bombyx mori* affected 20E titer, resulting in the death of most silkworms in the pre pupal stage [23]. In the cockroach *Blattella germanica*, 6th instar nymphs injected with ds*BgE75* survived for up to 80 days and eventually died without molting into adults [24].

Prior studies have shown that *E75* plays an important role in the regulation of insect development and metamorphosis. In this study, a total of four full-length *E75* cDNAs were identified, cloned, and characterized from the English grain aphid, *S. avenae*. Temporal expression profiling and RNAi-based functional analysis confirm the essential role of *SaE75* in ecdysone signaling pathway, and offer a new target site for future research on the development of RNAi-based biopesticide to control *S. avenae*.

## 2. Materials and Methods

### 2.1. Aphid Sampling and Colony Maintenance

*Sitobion avenae* (F.) were collected from common wheat, *Triticum aestivum,* at Langfang, Hebei Province, China (39°30′42″ N, 116°36′7″ E) in 2018. Aphids were taken to the lab and reared on 15 cm-tall wheat seedlings under growth conditions of 20 °C, 60% RH, and 16:8 h L:D photoperiod. All subsequent experiments used clonal female progeny from the third generation (G3).

### 2.2. Cloning of SaE75 Gene and Sequence Analysis

A total of four *E75* sequences from the pea aphid, *Acyrthosiphon pisum*, were downloaded from the National Center for Biotechnology Information website (NCBI, http://www.ncbi.nlm.nih.gov, accessed on 24 June 2022) with accessions of XM_016802193.2, XM_008182066.3, XM_008182067.3, XM_008182068.3. Then, local BLASTn was used to blast queries against our *S. avenae* transcriptomes [25] using TBtools (v1.098769) [26]. As a result, a putative *S. avenae E75 X 2* sequence was identified. Then, the primers of putative *S. avenae E75X1*, *E75X3*, and *E75X4* sequences were obtained by referring to the corresponding sequences of *A. pisum ApE75*. All sequences were BLAST against the nr database and submitted to NCBI database. The primers used in this study (Table S1) were designed with Primer Premier 5.0 software (Sigma-Aldrich, St. Louis, MO, USA) and synthesized at Sangon Biotech (Shanghai, China).

Total RNA was extracted from eight whole body adults of *S. avenae* using TRIzol reagent (Ambion, Foster City, CA, USA) according to the manufacturer’s protocol, and total RNA quantity and integrity were estimated by using a NanoPhotometer^®^ N60 micro ultraviolet spectrophotometer (Implen, München, Germany) and 1% agarose gel electrophoresis analysis. Then, 1 μg total RNA was reverse-transcribed using a HiScript III 1st Strand cDNA Synthesis Kit (+gDNA wiper) (Vazyme, Nanjing, China) following the manufacturer’s instructions (TIANGEN, Beijing, China). Subsequently, the full-length cDNA of the *E75* isoforms was amplified using a 2 × Phanta Flash Master Mix (Dye Plus) (Vazyme). The PCR reaction was carried out under the following conditions: 98 °C denaturation for 30 s, then 35 cycles of 98 °C for 10 s, 55 °C for 30 s, and 72 °C for 60 s. The PCR reaction annealing temperatures for *SaE75X1* and *X4* were 60 °C, *X2* and *X3* were 55 °C, respectively. The amplified sequences were cloned into a pTOPO-T Simple Vector (Invitrogen, Waltham, MA, USA) and then transformed into the DH5α strain of *Escherichia coli* (CWBIO, Taizhou, China). The positive clones were used for Sanger sequencing (Sangon Biotech).

The open reading frame (ORF) and protein translation were predicted using the ExPASy-translate tool (https://web.expasy.org/translate/, accessed on 27 July 2022). Theoretical isoelectric point (pI) and molecular weight (MW) were calculated based upon the ORF using the Compute pI/Mw tool (http://web.expas y.org/compute_pi/, accessed on 27 July 2022). Similarity searches were performed with NCBI-BLAST (https://www.ncbi.nlm.nih.gov/, accessed on 27 July 2022). DNAMAN v5.2.2 was used for multiple analysis of homologous sequences. The phylogenetic tree was constructed using the neighbor-joining (NJ) method with the Poisson model, as implemented in MEGA 11.0 software. The gaps/missing data treatment by pairwise deletion and node support was assessed using a bootstrap procedure with 1000 replicates [27]. The full-length proteins sequences from different insect species in Table S2 were selected to construct the phylogenetic tree.

### 2.3. Temporal Differences in SaE75 Expression between Winged and Wingless Morphs

*SaE75* expression level was estimated using RT-qPCR. Six different developmental stages [pseudo embryo (Pe, n = 50), the 1st instar nymph (N1, n = 40), 2nd instar nymph (N2, n = 30), 3rd instar nymph (N3, n = 20), 4th instar nymph (N4, n = 15), and adult (Ad, n = 10)] from both winged and wingless aphids were collected as described in our previous study [25]. Then, total RNA of each sample was extracted separately using the TRIzol reagent (Ambion) as described above, and 1 μg total RNA from each sample was reverse-transcribed with gDNA removing by using a TIANScript cDNA Kit (TIANGEN) following the manufacturer’s instructions. The primers were designed according to the common region of four *SaE75* isoform sequences and *Helicase* (*HEL*) was selected as the reference gene [28] (Table S1). The relative standard curve of each primer is generated by 5-fold concentration gradient dilution of the cDNA template. Reactions were performed in a CFX Connect Real-Time PCR Detection System (Bio-Rad, Hercules, CA, USA). The presence of a peak in melting curve analysis is used to confirm gene specific amplification and to exclude the generation of primer dimers and non-specific amplification, using slope analysis to determine RT-qPCR efficiencies for each primer. Subsequent RT-qPCR was performed using a SuperReal PreMix Plus kit (TIANGEN, Beijing, China). Each sample was amplified with an initial 95 °C denaturation for 15 min, and then 40 cycles of 95 °C for 10 s, 60 °C for 30 s, followed by the melting curve. Relative *SaE75* expression levels were calculated based on cycle threshold (CT) values using the 2^−ΔΔCT^ method [29]. All reactions were assayed in three biological and technical replications.

### 2.4. Treatment with 20E Analogue

The 24 h postnatal nymphs were treated with 1 mmol/L 20E (Sigma, USA) solution for 8 min, and control aphids were treated with 4.5% ethanol solution for the same time period [30]. Each treatment was run with 30 aphids per replicate across three replicates (n = 90). Samples were collected 24 h post-20E treatment, and all pooled nymphs of each treatment were used to isolate the total RNA. *SaE75* expression level was estimated using RT-qPCR as described above.

### 2.5. In Vivo Characterization of SaE75 RNAi on Molting

The primers for ds*SaE75* were designed according to the common region of the four *SaE75* isoform sequences, and with T7 adaptor on 5′ end (Table S1). ds*SaE75* fragments containing T7 (417 bp) was amplified using 2 × Es Taq MasterMix (Dye) (CWBIO) with an initial 94 °C denaturation for 2 min, and then 35 cycles of 94 °C for 30 s, 60 °C for 30 s, 72 °C for 30 s, and a final 72 °C extension for 2 min. The products were cloned into a pTOPO-T Simple Vector (Invitrogen) and then transformed into the DH5α strain of *E. coli* (CWBIO). The plasmid was extracted and verified via Sanger sequencing (Sangon Biotech). Then, the verified plasmid was used as the template for dsRNA synthesis by using a T7 High Yield RNA Transcription Kit following the manufacturer’s instructions (Vazyme). The *green fluorescent protein* (*GFP*) with T7 (464 bp) was used as negative control and was synthesized from *GFP* plasmid with methods described above.

The ds*SaE75* delivery system was prepared by mixing ds*SaE75* with nanocarrier SPc (A star polycation provided by Dr. Jie Shen from China Agricultural University) [31] and a 0.5% volume of detergent APG0810 (JiangSu WanQi Biotechnology Co., Ltd., Nantong, China) (surfactant and softened water). The final concentration for both SPc and dsRNA was 1 μg/μL [31,32]. The *GFP* and the nanocarrier SPc/detergent were used as the controls, while 0.1 μL of the ds*SaE75*/nanocarrier/detergent, ds*GFP*/nanocarrier/detergent, or nanocarrier/detergent (control) mixture was applied to the notum of a 24 h postnatal wingless nymph using a microinjector (Hamilton, Hamburg, Germany). Each experiment was run with 30 aphids per replicate across 6 replicates (n = 180). Three replicates were used for recording morphological defects and mortality daily until aphids reached adulthood. The other three replicates were collected 24 h after treatment; then, the relative expression levels of *SaE75* and its downstream genes, including *SaHr3* (hormone receptor like in 46), *Sabr-c* (broad-complex core protein gene), and *Saftz-f1* (*transcription factor 1*), were estimated and analyzed as described above. The primers amplification efficiencies for RT-qPCR ranged from 93.7 to 103.4% (Figure S1).

### 2.6. Statistical Analysis

All statistical analyses were carried out using SPSS 25.0 (SPSS Inc., Chicago, IL, USA). The data are presented as the mean ± standard error. Independent One-Way ANOVA followed by Tukey’s tests were used to detect significant differences in phenotype (winged vs. wingless) at each development stage, treatments with 20E, proportional mortality and expression quantity (*SaE75* and corresponding downstream gene) between treatments and corresponding NC, respectively. The significance threshold was set at *p* < 0.05.

## 3. Results

### 3.1. Molecular Cloning and Characterization of SaE75

**Figure 1:**
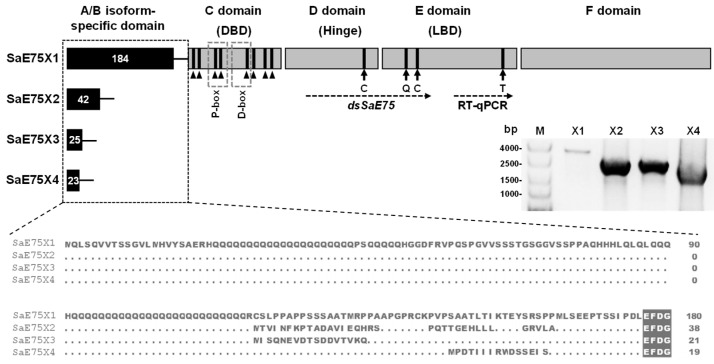
Structural analysis of four isoforms of *S. avenae SaE75*. The number of amino acids of each domain is indicated. The specific A/B domain with a different N-terminal sequence is illustrated in a black dotted box, and the different sequence alignments are shown. The C domain (DNA-binding domain, DBD), D domain (Hinge), E domain (ligand-binding domain, LBD), and F domain are highlighted in gray rectangles, which all have the same amino acid sequences among the four isoforms. The cysteine residues of the two zinc finger motifs are labeled by black triangles, and the P-box and D-box in the DNA-binding domain are illustrated in gray dotted boxes. The key amino acid residues involved in heme binding are labeled by arrows. The long-dotted arrow indicates the primer amplification region for RT-qPCR and ds*SaE75*. Electrophoretic analysis of full-length sequences of the four SaE75 isoforms is shown in the right middle region. M: DL 15,000 Plus marker (Beijing Zoman Biotechnology Co., Ltd., Beijing, China).

A putative 2625 bp cDNA sequence of S. avenae was identified based on the four isoforms of A. pisum ApE75 using local BLASTn with our own transcriptome data [25]. Then, the full-length sequence of S. avenae E75 was cloned and named SaE75 X 2. The other putative isoforms of S. avenae SaE75 were also cloned and named SaE75 X 1, SaE75 X 3 and SaE75 X 4 (Figure 1). The length of the four isoforms X1–X4 was 3048 bp, 2625 bp, 2505 bp, and 2179 bp, and encoded 1015, 874, 856, and 835 amino acids, respectively. The relative molecular weights were 112.26 kD, 96.33 kD, 94.53 kD, and 92.66 kD, respectively. The isoelectric points were 8.76, 8.69, 8.50, and 8.64, respectively. The similarity of four SaE75 isoforms is 83.85% based on their non-conserved N terminal region (Figure 1). Four full-length sequences of SaE75 X 1–4 were submitted to the NCBI database with accessions of OP058107 to OP058110 (Table S2). The sequence alignments of the four SaE75 and four ApE75 isoforms showed 95.76%, 98.18, 98.02%, and 98.14% identity, respectively (Figure S1). All four SaE75 isoforms showed typical nuclear receptor domains, including an A/B isoform-specific domain, a C domain (DNA-binding domain, DBD), a D Domain (Hinge domain), an E domain (ligand-binding domain, LBD), and an F domain. The A/B domains (N-terminal domain) showed different sequences and the C to F domains (C-terminal domain) showed mostly similar sequences and are often called common domains. The C domain contained two zinc finger regions, and each zinc finger region had four cysteine residues. The E domain contained important amino acid residues that participate in heme binding, as well as P-box and D-box regions that regulate DNA recognition (Figure 1 and Figure S1).

### 3.2. Phylogenetic Analysis

**Figure 2:**
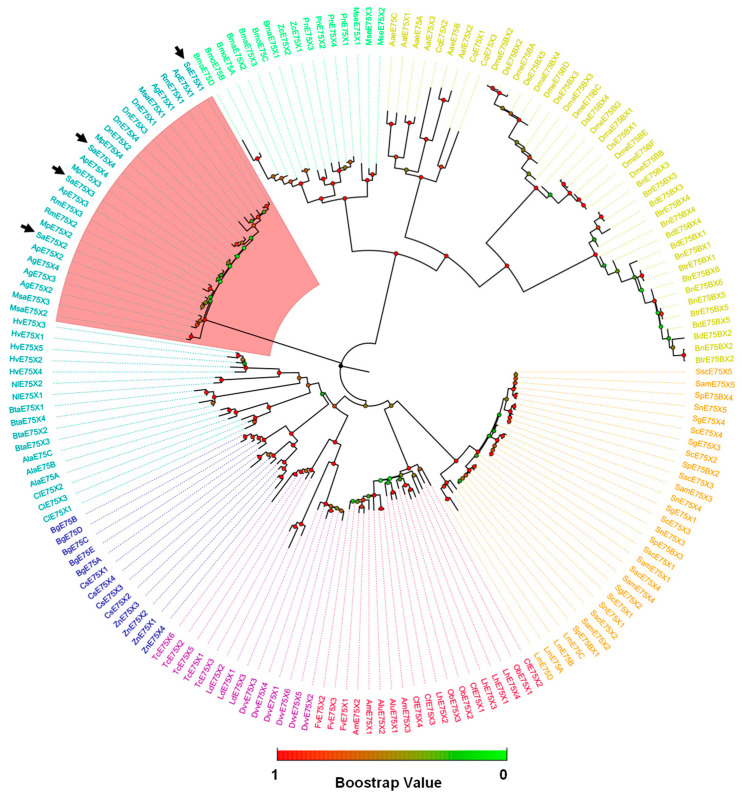
Phylogenetic analysis of E75 from different insect species. The cyan shows Hemiptera; the blue shows Blattaria; the purple shows Coleoptera; the red shows Hymenoptera; the orange shows Orthoptera; the yellow shows Diptera; the green shows Lepidoptera; and arrows show the four SaE75 isoforms. Ap: *Acyrthosiphon pisum*; Ag: *Aphis gossypii*; Dn: *Diuraphis noxia*; Msa: *Melanaphis sacchari*; Mp: *Myzus persicae*; Rm: *Rhopalosiphum maidis*; Sa: *Sitobion avenae*; Alu: *Apolygus lucorum*; Bta: *Bemisia tabaci*; Cl: *Cimex lectularius*; Hv: *Homalodisca vitripennis*; Nl: *Nilaparvata lugens*; Bg: *Blattella germanica*; Cs: *Cryptotermes secundus*; Zn: *Zootermopsis nevadensis*; Dvv: *Diabrotica virgifera virgifera*; Ld: *Leptinotarsa decemlineata*; Tc: *Tribolium castaneum*; Ala: *Apis laboriosa*; Am: *Apis mellifera*; Fv: *Frieseomelitta varia*; Cf: *Camponotus floridanus*; Lh: *Linepithema humile*; Ob: *Ooceraea biroi*; Sg: *Schistocerca gregaria*; Sc: *Schistocerca cancellate*; Sam: *Schistocerca americana*; Ssc: *Schistocerca serialis cubense*; Sp: *Schistocerca piceifrons*; Sn: *Schistocerca nitens*; Lm: *Locusta migratoria*; Aae: *Aedes aegypti*; Aal: *Aedes albopictus*; Cq: *Culex quinquefasciatus*; Bn: *Bactrocera neohumeralis*; Btr: *Bactrocera tryoni*; Bd: *Bactrocera dorsalis*; Dma: *Drosophila mauritiana*; Ds: *Drosophila simulans*; Dme: *Drosophila melanogaster*; Bmo: *Bombyx mori*; Bma: *Bombyx mandarina*; Mse: *Manduca sexta*; Pn: *Pieris napi*; Zc: *Zerene cesonia*.

A total of 178 E75 protein from 45 species in 7 orders were selected to construct the phylogenetic tree (Table S2). The result showed that the closest relative of *S. avenae* SaE75 is *A. pisum* ApE75. Interestingly, the Hemiptera insects E75 were divide into two clades, one for Aphididae, and another for the other Hemiptera insects, *Apolygus lucorum*, *Bemisia tabaci*, *Cimex lectularius*, *Homalodisca vitripennis*, and *Nilaparvata lugens* (Figure 2). All the aphid E75s were clustered into the same clade, which included *A. pisum*, *Aphis gossypii*, *Diuraphis noxia*, *Melanaphis sacchari*, *Myzus persicae*, and *Rhopalosiphum maidis*.

### 3.3. Expression Characteristics of SaE75

**Figure 3:**
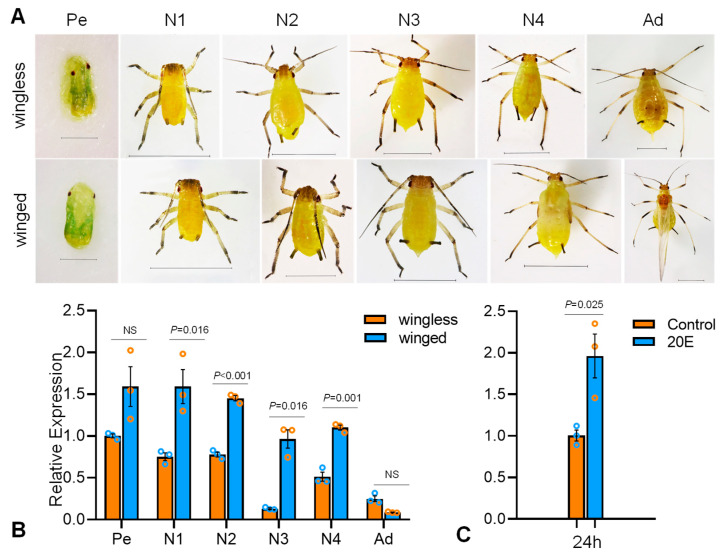
Temporal dynamics of *SaE75*. (**A**) Developmental profiles of two morphs of *S. avenae*. Relative abundance of *SaE75* on (**B**) different developmental stages and (**C**) 20E treatment were validated using RT-qPCR. Pe: pseudo embryo; N1: nymph 1; N2: nymph 2; N3: nymph 3; N4: nymph 4; Ad: adult; Scale bar: 20 μm for Pe; 100 μm for N1-Ad. Different color circle represents three replications of experiment. Error bars represent means ± SD. Data were normalized with respect to *helicase* (*HEL*), and bar graphs were fit to the data using GraphPad Prism (v8.0.1). The *p* values indicate significant differences, and NS means not significant.

Samples from six different developmental stages (Figure 3A), including Pe, N1, N2, N3, N4, and Ad, were collected from both winged and wingless *S. avenae* to detect expression levels of *SaE75* using RT-qPCR. The result showed that the relative expression levels of *SaE75* had a similar trend from Pe to Ad within both winged and wingless morphs (Figure 3B). Within morphs, *SaE75* was expressed at relatively higher levels in the Pe and N1 stages, and expression subsequently decreased across later stages except for an increase from N3 to N4. Among all developmental stages, expression was the lowest in winged Ad, which was approximately 18-fold lower than that for winged Pe and N1. *SaE75* in wingless N3 and Ad had relatively low levels of expression, which was approximately 7.6- and 4-fold lower than that for wingless Pe, respectively. The expression of *SaE75* showed significant differences between winged and wingless morphs at N1 (t_N1_ = −4.021, *p* = 0.016), N2 (t_N2_ = −15.855, *p* = 0.000), N3 (t_N3_ = −7.608, *p* = 0.016), and N4 (t_N4_ = −9.387, *p* = 0.001) (Figure 3B). The 24 h postnatal nymphs were treated with 1 mmol/L 20E; after 24 h of treatment, the sample was collected to detect the expression of *SaE75*. The result showed that *SaE75* expression level was significantly increased to 1.96-fold control levels (t_20E_ = −3.513, *p* = 0.025; Figure 3C).

### 3.4. In Vivo Validation of SaE75 Impacts on Molting

**Figure 4:**
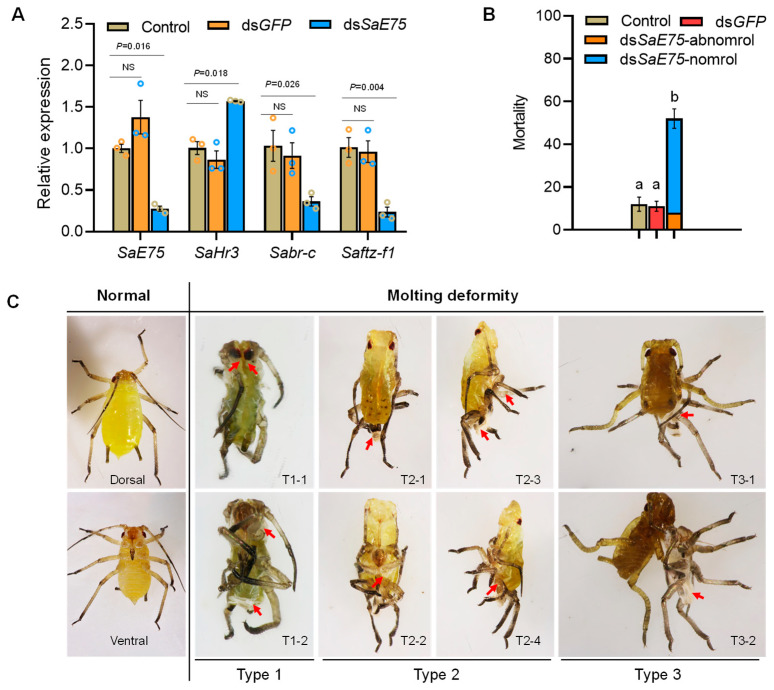
Functional analysis of *SaE75*. Data shown for ds*SaE75* with respect to (**A**) the expression level of *SaE75* and downstream genes (*Sabr-c*, *SaHr3* and *Saftz-f1*), and (**B**) mortality. (**C**) Phenotypic impacts of ds*SaE75* on *S. avenae* molting. ds*SaE75*-normal: aphids dead without any detectable morphological abnormality; ds*SaE75*-abnormal: aphids dead with molting deformed; T1-1 and T1-2: dorsal view and ventral view of “unable to molt” phenotype; T2-1 to T2-4: dorsal view, ventral view, and side view of “partial molt” phenotype; T3-1 and T3-2: dorsal view and ventral view of “almost finished molt” phenotype. Red arrow shows the position of molting deformity. Expression differences of four genes among control, ds*GFP*, and ds*SaE75* groups were tested using one-way ANOVA followed by Tukey’s tests. Different color circle represents three replications of experiment. Different letters (a, b) indicate significant differences between means (*p* < 0.05). Red arrows represent the location of abnormal molting. Error bars represent means ± SD. The *p* values indicate significant differences, and NS means not significant.

The introduction of ds*SaE75* into *S. avenae* nymphs influenced relative gene expression and induced high rates of mortality and molting deformity (Figure 4). Expression of *SaE75* was significantly inhibited and decreased by 72% compared with the control group (t*_SaE75_* = 12.555, *p* = 0.016; Figure 4A). Furthermore, the expression of the downstream ecdysone pathway gene *SaHr3* was significantly up-regulated by 57%, while *Sabr-c* and *Saftz-f1* were significantly down-regulated by 64% and 76%, respectively (t*_SaHr3_* = −7.258, *p* = 0.018; t*_Sabr-c_* = 3.459, *p* = 0.026; t*_Saftz-f1_* = 5.863, *p* = 0.004; Figure 4A).

The mortality of aphid in the dsRNA treatment groups (51%) was significantly greater than the mortality in the negative control groups ds*GFP* (11%) and H_2_O (12%) (F = 27.41, *p* < 0.001, Figure 4B), within which, 8% aphid could not molt normally and showed three types of molting deformity: (Type 1) unable to molt; (Type 2) partial molt; and (Type 3) almost finished molt (Figure 4C); 43% aphid died without obvious special characteristics. All the aphids that died were in the second or third instar nymph stages, and could not develop into adults. The remaining surviving aphids in the dsRNA treatment groups all reach adulthood in 11.07 ± 0.15 days; the remaining surviving aphids in the control and ds*GFP* treatment groups all reached adulthood in 10.34 ± 0.12 days and 10.52 ± 0.13 days, respectively (*X*^2^ = 11.561, *df* = 2, *p* = 0.003). After the surviving aphids reached the adulthood, the rate of winged morphs was 29.93, 26.67, and 18.89% in the control, ds*GFP*, and ds*SaE75* treatments (F = 1.295, *p* = 0.341, Figure S2), respectively.

## 4. Discussion

The number of *E75* isomers varies considerably across insect species due to alternative splicing. For example, there are five isoforms of *E75* in *Tribolium castaneum* and *B. germanica* [24,33], four isoforms of *ApE75* in *A. pisum* [13], and three isoforms in *B. mori* [23]. In this study, we cloned and characterized four *SaE75* isoforms of *S. avenae*. The amino acid sequences within the C to F domains were found to share 98.22% similarity to the four *A. pisum E75* isoforms. The C domain (DBD) contains two C4 zinc finger structures, which may participate in the binding of zinc atoms to the DNA sequences of hormone response elements [34]. Previous studies suggest that *Drosophila* E75s function as heme sensors, and their heme centers are located in the D (Hinge) and E domains (LBD), which contain four key amino acid residues binding to diatomic molecules, such as CO and NO [35]. Analogously, the four cysteine residues involved in heme binding are conserved in the four isoforms of *S. avenae* E75. Furthermore, the P-box and D-box in the DBD region, which determine the specificity of DNA binding and the formation of homodimers, respectively, is also highly conserved, suggesting that the nuclear receptor gene *E75* in insects is relatively conserved.

Our phylogenetic tree showed that the E75 sequences were clustered corresponding to different orders except for Hemipteran insects, which is consistent with E75 clustering results in other insects, such as the Hemipteran *A. lucorum* [36] and Orthoptera *Locusta migratoria* [37]. Hemipteran insects E75 were separated into two branches, and all the aphid E75s were clustered into the same clade, and the closest relative was *A. pisum* ApE75. We speculate that these genes are homologous genes that are evolutionarily conserved and have similar biological functions.

The role of 20E in inducing insect molting and metamorphosis is well-characterized [10,38]. *E75* is an early regulated gene that is involved in many physiological processes within the insect ecdysone signaling pathway. Previous studies have shown that most insect *E75s* are highly expressed in the early instars, suggesting that they are involved in regulation of the ecdysone signaling pathway [13,23,33]. In our study, *SaE75* was expressed most highly in the Pe and N1 stages, and then subsequently decreased across later stages except for an increase from N3 to N4, both in winged and wingless aphids. This result suggests that the first instar may be an important developmental stage for *SaE75* to regulate ecdysone signaling pathway in aphids. In *N. lugens*, it was also found that *NlE75* expression increased from the egg to the first instar nymph stages, and subsequently decreased across later stages except for an increase from day 1 to day 3 of the fifth instar nymph stage [39].

After treatment with 20E, the expression level of *SaE75* was significantly up-regulated, consistent with reports in other insects. For example, in *Aedes aegypti*, in vitro fat body culture showed that 20E could induce the expression of all isoforms of *AaE75* [40]. In the early fifth instar *B. mori* larvae, three E75 isoforms also showed responses to 20E at the mRNA and protein levels [23]. In *B. germanica*, anatomical fat bodies were cultured in vitro, and the 20E that was added induced the expression of four isoforms of *BgE75* (*BgE75A*, *BgE75B*, *BgE75C*, and *BgE75E*) [24].

In this study, the common regions of four *SaE75* isoforms were selected to design ds*SaE75* and perform in vivo RNAi to affect the molting process of *S. avenae*, resulting in abnormal molting and nymphs’ high mortality, failing to molt to adult and finally died with or without morphological abnormality. As previously demonstrated in *N. lugens*, specific RNAi isoforms can silence the expression of the corresponding target gene, but only silencing achieved using dsRNA designed to target the common region of these isoforms can affect the molting process, making the third instar nymphs unable to molt and die, and the fifth instar nymphs die during the molting process [39]. In *B. germanica*, RNAi targeting the common region of *BgE75* isoforms inhibited molting in sixth-instar nymphs and showed variable degree of duplication of the ectodermic-derived structures [24]. Similarly, *L. decemlineata LdE75* RNAi larvae cannot molt into adult normally and died with their larval cuticle still wrapped [22]. *D. melanogaster* larvae with all *E75* isoforms mutation remained in the first instar, failed to molt to the second instar, and finally died without any detectable morphological abnormality [18]. These homologous phenotypes amongst various insect species suggest that *E75* may play an identical role in insect molting and developmental process.

Development is essential, especially for most hemimetabolous insects; growth and maturation occur simultaneously throughout successively nymphal stages, and ecdysone signaling pathway is involved in this development process. A previous study showed that *BgE75* is necessary to nymphal–nymphal and nymphal–adult transition in *B. germanica* [24]. The results presented herein by using RNAi in vivo during the first nymphal instar of *S. avenae* indicate that many individuals died in the second or third instar nymph stages and could not develop to adults. After the surviving aphids reaching adulthood, we found that ds*SaE75* treatment prolonged the developmental period, but the winged rate had no significant differences among each treatment. Given that the resultant winged morphs all maintained normal wings, we hypothesized that *E75* is primarily associated with molting and development rather than wing formation in *S. avenae*. Furthermore, in the ecdysone signaling pathway, *br-c* is an early response gene, *Hr3* is an early and late response gene, and *ftz-f1* is a late response gene [13]. In this study, an array of genes associated with the ecdysone signal pathway has been affected by *SaE75* RNAi, and similar results were found in other insects. *Drosophila DHR3* induces the expression of *Dftz-f1*, while *DE75B* together with *DHR3* leads to the down-regulation of *Dftz-f1* expression [35]. In *B. mori*, *BmHr3A* interacted with *BmE75*, and *BmE75* inhibited the expression of *BmHr3* [41]. In addition, *LdE75* RNAi in *L. decemlineata* inhibited the expression of *Ldftz-f1*, while ingestion of an ecdysteroid agonist, halofenozide (Hal), induced *Ldftz-f1* expression, suggesting that *LdE75* is required for *Ldftz-f1* induction [22]. Taken together, *SaE75* participates in the development of *S. avenae* through the ecdysone signal pathway.

## Data Availability

The data presented in this study are available in the article.

## Supplementary Materials

The following supporting information can be downloaded at: https://www.mdpi.com/article/10.3390/insects14040383/s1, Figure S1: Multiple alignment of amino acid sequence characteristics of E75 isoforms between *S. avenae* and *A. pisum*; Figure S2: Functional analysis of *SaE75* on *S. avenae* wing development. Table S1: Primer information in this study; Table S2: Species information for phylogenetic tree used in this study.
