# Supplementary material for: Functional Characterization of the Nuclear Receptor Gene SaE75 in the Grain Aphid, Sitobion avenae
Source: Insects. 2023 Apr 14;14(4):383. doi: 10.3390/insects14040383 (PMC10144623; doi:10.3390/insects14040383)
Supplement: Supplementary file 1 [file insects-14-00383-s001.zip › insects-2291883-supplementary.pdf]

Supplementary materials

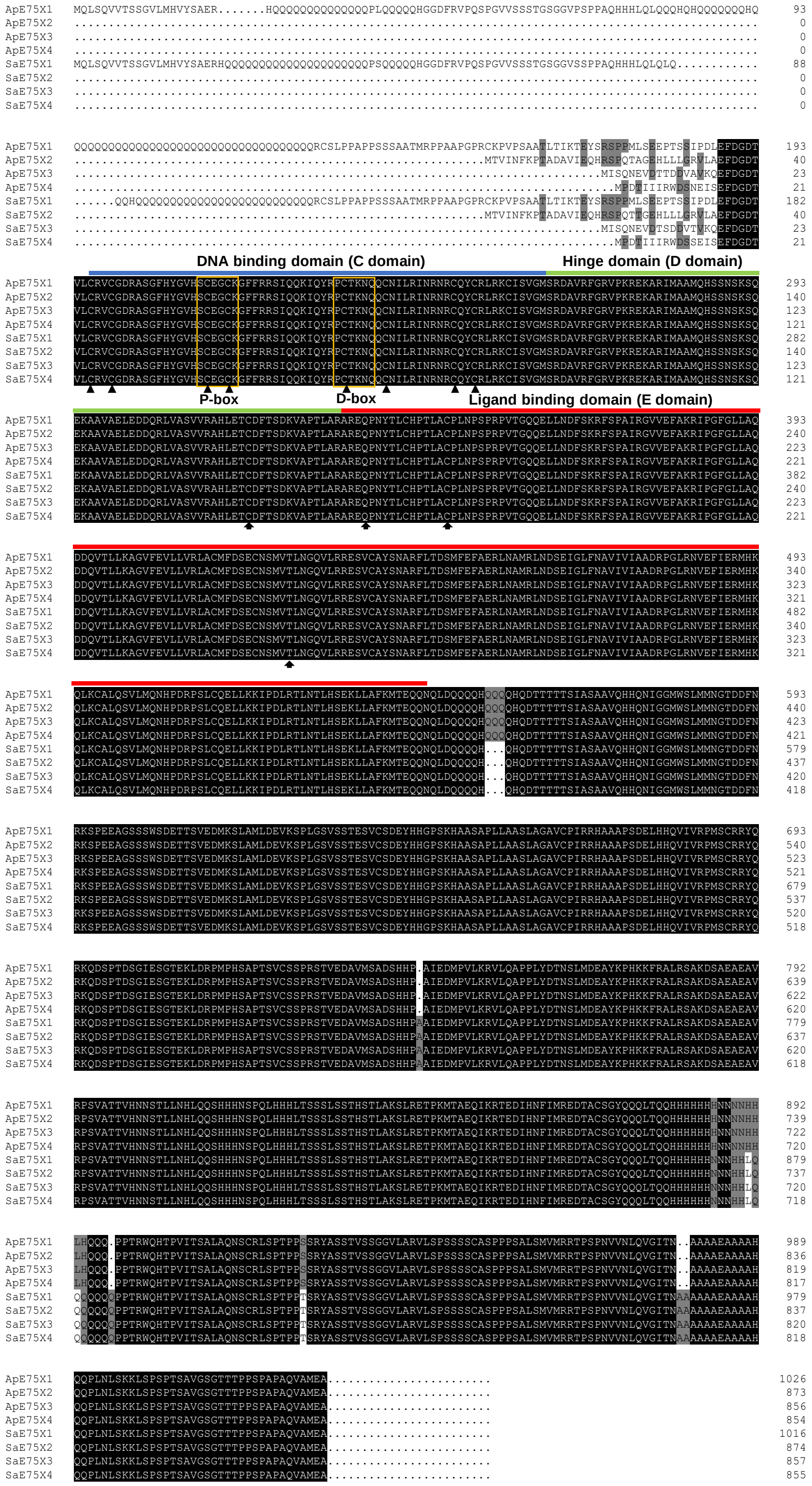

Figure S1. Multiple alignment of amino acid sequence characteristics of E75 isoforms between *S. avenae* and *A. pisum*. The DNA binding domain (C domain), the hinge domain (D domain), and the ligand binding domain (E domain) are tagged above the sequence with blue, green, and red lines, respectively; the black triangles indicate that there are four Cys residues in the zinc finger, and the important amino acid residues involved in heme binding are marked with the black arrow.

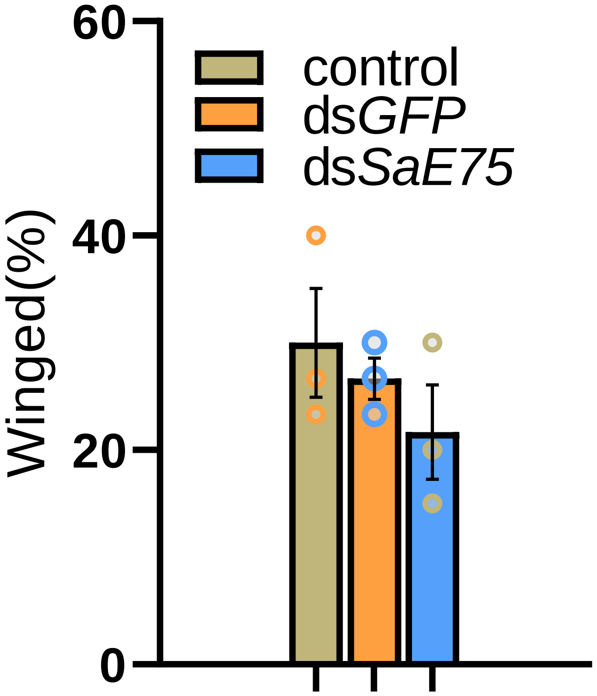

Figure S2. Functional analysis of *SaE75* on *S. avenae* wing development. Different color circle represents three replications of experiment. Error bars represent means ± SD.

Tables

Table S1. Primer information in this study

Gene	Primer sequences	Application	Length of product (bp)	Amplification efficiency
SaE75X1 F	ATGCAGTTGTCTCAAGTCGTA	cDNA cloning	3048	-
SaE75X1 R	TTACGCTTCCATGGCCACCTGAGCG			
SaE75X2 F	TTACGCTTCCATGGCCACC		2625	-
SaE75X2 R	ATGACCGTTATAAATTTCAA			
SaE75X3 F	ATGATTAGTCAAAACGAAGTC		2571	-
SaE75X3 R	ATGGCCACCTGAGCGGGAGCA			
SaE75X4 F	ATGCCAGACACGATAATTATC		2505	-
SaE75X4 R	ACGGCGGACGTCGGCGACGGGGACA			
**SaE75*F	CGACTCTGAGTGTAACAGCA	RT-qPCR	165	103.4%
**SaE75*R	GCGTTGAATAGACCAATTTC			
*Saftz-f1*F	CAGACATAATCCTCCGTCAT		194	93.7%
*Saftz-f1*R	GTCTTGGCTGTATTGGTGAT			
*Sabr-c*F	CTGAGAGACGATGAGGACTT		199	97.3%
*Sabr-c*R	CGTGGTAGATGAACTCAACC			
*SaHr3*F	GTCCCGACATTATGACCTAC		151	96.9%
*SaHr3*R	CAGTAAGTTTCAACGAAGCC			
*HEL*F	TGCTACCGGATGTGGGAAAA	RT-qPCR (Reference gene)	152	103.0%
*HEL*R	TCCAGCCACGTTCTCTGTTT			
*ds*SaE75* F	taatacgactcactatagggGGTCTATTCAACGCCGTCAT	RNAi	377	-
*ds*SaE75* R	taatacgactcactatagggGCGAAGTCGAACATGGAGTC			
ds*GFP*F	taatacgactcactatagggCCTGTTCCATGGCCAAC	RNAi	424	-
ds*GFP*R	taatacgactcactatagggAAAGGACAGGGCCATCG			

Note: * means the primers were designed from the common region of *SaE75X1-X4*; sequences of T7 promoter in primers were lowercase alphabet.

Table S2. Species information for phylogenetic tree used in this study

Order	Species	Gene name	GenBank accession number
Hemiptera	*Acyrthosiphon pisum*	*ApE75X1, ApE75X2, ApE75X3, ApE75X4*	XP_016657682.1, XP_008180288.1, XP_008180289.1, XP_008180290.1
	Aphis gossypii	*AgE75X1, AgE75X2, AgE75X3, AgE75X4*	XP_050054422.1, XP_050054429.1, XP_050054432.1, XP_050054438.1
	Diuraphis noxia	*DnE75X1, DnE75X2, DnE75X3, DnE75X4*	XP_015372971.1, XP_015372972.1, XP_015372973., XP_015372974.1
	Melanaphis sacchari	*MsaE75X1, MsaE75X2, MsaE75X3*	XP_025206796.1, XP_025206797.1, XP_025206798.1
	Myzus persicae	*MpE75X1, MpE75X2, MpE75X3, MpE75X4*	XP_022162355.1, XP_022162356.1, XP_022162357.1, XP_022162358.1
	Rhopalosiphum maidis	*RmE75X1, RmE75X2, RmE75X3*	XP_026814295.1, XP_026814296.1, XP_026814297.1
	Sitobion avenae	*SaE75X1, SaE75X2, SaE75X3, SaE75X4*	OP058107, OP058108, OP058109, OP058110
	Apolygus lucorum	*AluE75A, AluE75B, AluE75C*	ATN39778.1, KAF6203537.1, ATN39780.1
	Bemisia tabaci	*BtaE75X1, BtaE75X2, BtaE75X3, BtaE75X4*	XP_018907601.1, XP_018907602.1, XP_018907603.1, XP_018907604.1
	Cimex lectularius	*ClE75X1, ClE75X2, ClE75X3*	XP_014240379.1, XP_014240380.1, XP_014240381.1
	Homalodisca vitripennis	*HvE75X1, HvE75X2, HvE75X3, HvE75X4, HvE75X5*	XP_046680981.1, XP_046680982.1, XP_046680983.1, XP_046680984.1, XP_046680985.1
	Nilaparvata lugens	*NlE75X1, NlE75X2*	XP_039292205.1, AST48086.1
Blattodea	*Blattella germanica*	*BgE75A, BgE75B, BgE75C, BgE75D, BgE75E*	CAJ87513.1, CAJ87514.1, CAM97373.1, CAM97374.1, CAM97375.1
	Cryptotermes secundus	*CsE75X1, CsE75X2, CsE75X3, CsE75X4*	XP_023709200.1, XP_023709201.2, XP_023709202.1, XP_023709203.1
	Zootermopsis nevadensis	*ZnE75X1, ZnE75X2, ZnE75X3, ZnE75X4*	XP_021938067.1, XP_021938068.1, XP_021938069 .1, XP_021938070.1
Coleoptera	*Diabrotica virgifera virgifera*	*DvvE75X1, DvvE75X2, DvvE75X3, DvvE75X4, DvvE75X5, DvvE75X6*	XP_028130296.1, XP_028130297.1, XP_028130298.1, XP_028130301.1, XP_028130302.1, XP_028130303.1
	Leptinotarsa decemlineata	*LdE75X1, LdE75X2, LdE75X3*	AKN56577.1, AKN56578.1, ALU57795.1
	Tribolium castaneum	*TcE75X1, TcE75X2, TcE75X3, TcE75X4, TcE75X5*	NP_001308599.1.1, XP_008197844, XP_971362.2, XP_015838721, XP_015838722.1
Hymenoptera	*Apis laboriosa*	*AlaE75X1, AlaE75X2*	XP_043795752.1, XP_043795762.1
	Apis mellifera	*AmE75X1, AmE75X2, AmE75X3*	XP_006564381.2, XP_006564382.1, XP_006564383.2
	Frieseomelitta varia	*FvE75X1, FvE75X2, FvE75X3*	XP_043510979.1, XP_043510980.1, XP_043510981.1
	Camponotus floridanus	*CfE75X1, CfE75X2, CfE75X3, CfE75X4*	XP_025268006.1, XP_025268007.1, XP_011259848.2, XP_011259847.2
	Linepithema humile	*LhE75X1, LhE75X2, LhE75X3, LhE75X4*	XP_012226918.1, XP_012226919.1, XP_012226920.1, XP_012226921.1
	Ooceraea biroi	*ObE75X1, ObE75X2, ObE75X3*	XP_011332916.2, XP_019886380.2, XP_011332918.2
Orthoptera	*Schistocerca gregaria*	*SgE75X1, SgE75X2, SgE75X3, SgE75X4*	XP_049859092.1, XP_049859093.1, XP_049859094.1, XP_049859095.1
	Schistocerca cancellate	*ScE75X1, ScE75X2, ScE75X3, ScE75X4*	XP_049778571.1, XP_049778572.1, XP_049778573.1, XP_049778574.1
	Schistocerca americana	*SamE75X1, SamE75X2, SamE75X3, SamE75X4,*	XP_046992523.1, XP_046992524.1, XP_046992525.1, XP_046992527.1

		SamE75X5	
	Schistocerca serialis cubense	*SscE75X1, SscE75X2, SscE75X3, SscE75X4, SscE75X5*	XP_049956056.1, XP_049956057.1, XP_049956059.1, XP_049956060.1, XP_049956061.1
	Schistocerca piceifrons	*SpE75X1, SpE75X2, SpE75X3, SpE75X4, SpE75X5*	XP_047110316.1, XP_047110317.1, XP_047110319.1, XP_047110320.1
	Schistocerca nitens	*SnE75X1, SnE75X2, SnE75X3, SnE75X4, SnE75X5*	XP_049807890.1, XP_049807891.1, XP_049807892.1, XP_049807893.1, XP_049807894.1
	Locusta migratoria	*LmE75X1, LmE75X2, LmE75X3*	QKY88966.1, QKY88967.1, QKY88968.1
Diptera	*Aedes aegypti*	*AaeE75A, AaeE75B, AaeE75C*	CAL36974.1, CAL36973.1, CAL36975.1
	Aedes albopictus	*AalE75X1, AalE75X2, AalE75X3*	XP_029720431.1, XP_019556691.1, XP_019556692.1
	Culex quinquefasciatus	*CqE75X1, CqE75X2, CqE75X3*	XP_038113830.1, XP_001849917.2, XP_038113837.1
	Bactrocera neohumeralis	*BnE75BX1, BnE75BX2, BnE75BX3, BnE75BX4, BnE7B5X5, BnE75BX6*	XP_050336968.1, XP_050336969.1, XP_050336970.1, XP_050336971.1, XP_050336972.1, XP_050336973.1
	Bactrocera tryoni	*BtrE75BX1, BtrE75BX2, BtrE75BX3, BtrE75BX4, BtrE75BX5, BtrE75BX6*	XP_039965865.1, XP_039965866.1, XP_039965868.1, XP_039965869.1, XP_039965870.1, XP_039965871.1
	Bactrocera dorsalis	*BdE75BX1, BdE75BX2, BdE75BX3, BdE75BX4, BdE75BX5*	XP_049315304.1, XP_049315306.1, XP_049315307.1, XP_049315308.1, XP_049315309.1
	Drosophila mauritiana	*DmaE75BX1, DmaE75BX2, DmaE75BX3, DmaE75BX4*	XP_033157614.1, XP_033157615.1, XP_033157616.1, XP_033157617.1
	Drosophila simulans	*DsE75BX1, DsE75BX2, DsE75BX3, DsE75BX4, DsE75BX5*	XP_039149874.1, XP_016032380.2, XP_039149875.2, XP_039149876.1, XP_016032381.2
	Drosophila melanogaster	*DmeE75BA, DmeE75BB, DmeE75BC, DmeE75BD, DmeE75BE, DmeE75BF, DmeE75BG*	NP_524133.2, NP_730321.1, NP_730322.1, NP_730323.1, NP_001246821.1, NP_001246822.1, NP_001303380.1
Lepidoptera	*Bombyx mori*	*BmoE75A, BmoE75B, BmoE75C*	NP_001106079.1, NP_001106080.1, NP_001037042.1
	Bombyx mandarina	*BmaE75X1, BmaE75X2, BmaE75X3*	XP_028027338.1, XP_028027339.1, XP_028027340.1
	Manduca sexta	*MseE75X1, MseE75X2, MseE75X3*	XP_030039289.1, XP_030039296.1, XP_030039301.1
	Pieris napi	*PnE75X1, PnE75X2, PnE75X3, PnE75X4, PnE75X5*	XP_047509890.1, XP_047509891.1, XP_047509892.1, XP_047509893.1
	Zerene cesonia	*ZcE75X1, ZcaE75X2*	XP_038215405.1, XP_038215406.1
